# X-Box Binding Protein 1 Is Essential for the Anti-Oxidant Defense and Cell Survival in the Retinal Pigment Epithelium

**DOI:** 10.1371/journal.pone.0038616

**Published:** 2012-06-08

**Authors:** Yimin Zhong, Jingming Li, Joshua J. Wang, Chen Chen, Julie-Thu A. Tran, Anisse Saadi, Qiang Yu, Yun-zheng Le, Md Nawajes A. Mandal, Robert E. Anderson, Sarah X. Zhang

**Affiliations:** 1 Department of Medicine, Section of Endocrinology and Diabetes, University of Oklahoma Health Sciences Center, Oklahoma City, Oklahoma, United States of America; 2 Harold Hamm Diabetes Center at University of Oklahoma, Oklahoma City, Oklahoma, United States of America; 3 Department of Ophthalmology, University of Oklahoma Health Sciences Center, Oklahoma City, Oklahoma, United States of America; 4 Dean A. McGee Eye Institute, Oklahoma City, Oklahoma, United States of America; 5 Department of Cell Biology, University of Oklahoma Health Sciences Center, Oklahoma City, Oklahoma, United States of America; 6 Oklahoma Center for Neuroscience, Oklahoma City, Oklahoma, United States of America; 7 State Key Laboratory of Ophthalmology, Zhongshan Ophthalmic Center, Sun Yat-sen University, Guangzhou, China; Johns Hopkins School of Medicine, United States of America

## Abstract

Damage to the retinal pigment epithelium (RPE) is an early event in the pathogenesis of age-related macular degeneration (AMD). X-box binding protein 1 (XBP1) is a key transcription factor that regulates endoplasmic reticulum (ER) homeostasis and cell survival. This study aimed to delineate the role of endogenous XBP1 in the RPE. Our results show that in a rat model of light-induced retinal degeneration, XBP1 activation was suppressed in the RPE/choroid complex, accompanied by decreased anti-oxidant genes and increased oxidative stress. Knockdown of XBP1 by siRNA resulted in reduced expression of SOD1, SOD2, catalase, and glutathione synthase and sensitized RPE cells to oxidative damage. Using Cre/LoxP system, we generated a mouse line that lacks XBP1 only in RPE cells. Compared to wildtype littermates, RPE-XBP1 KO mice expressed less SOD1, SOD2, and catalase in the RPE, and had increased oxidative stress. At age 3 months and older, these mice exhibited apoptosis of RPE cells, decreased number of cone photoreceptors, shortened photoreceptor outer segment, reduced ONL thickness, and deficit in retinal function. Electron microscopy showed abnormal ultrastructure, Bruch's membrane thickening, and disrupted basal membrane infolding in XBP1-deficient RPE. These results indicate that XBP1 is an important gene involved in regulation of the anti-oxidant defense in the RPE, and that impaired activation of XBP1 may contribute to RPE dysfunction and cell death during retinal degeneration and AMD.

## Introduction

Age-related macular degeneration (AMD) is the leading cause of blindness in the elderly. Approximately 1.47% of the US adults aged 40 years and older are currently affected by AMD, and this number will increase dramatically by 2020 [1]. The dry form of AMD, characterized by depigmentation of the retinal pigment epithelial cells (RPE), loss of RPE cells, and drusen formation, presents in 80–90% of the AMD patients. Dry AMD may progresses to geographic atrophy or wet AMD leading to impairment of central vision in patients. Although the pathogenic mechanisms of AMD are not fully understood, compelling evidence suggests that decreased anti-oxidant defense with age in highly metabolically active retinal cells is a key etiological factor for AMD. In the macular RPE from donors older than 70 yrs, the level of metallothionein, a potent antioxidant, was decreased by 68% when compared to the younger donors [2]. The activity of catalase, an anti-oxidant enzyme, was also decreased with age [3]. These changes suggest that the RPE in the elderly may be more susceptible to oxidative damage. Indeed, mice deficient of superoxide dismutase 1 (SOD1), a major scavenger enzyme that removes superoxide (O_2_^−^), demonstrated accelerated AMD-like lesions in the retina, including drusen, thickening of Bruch's membrane, and choroidal neovascularization (CNV) [4]. In contrast, supplementation with anti-oxidant vitamins and zinc significantly reduced disease progression to advanced AMD in high-risk patients, which emphasizes the role of oxidative stress as a primary culprit in AMD [5], [6]. Although there is a controversy as to whether the RPE injury is an initial event resulting in photoreceptor loss in AMD, the critical role of RPE cells in supporting photoreceptor cell survival and function has been firmly established. Without normal RPE, photoreceptors will likely undergo apoptosis and cell death [7]. In human retinas with AMD, apoptotic photoreceptors were found clustered near the area of RPE atrophy, suggesting that loss of RPE cell may proceed photoreceptor apoptosis during disease development [8]. Further, down-regulation of SOD2, a major anti-oxidant enzyme in the mitochondria, in the RPE by a subretinal injection of an AAV-ribozyme-mediated knockdown of SOD2 mRNA in the RPE of wildtype mice resulted in hypopigmentation, lipofuscin accumulation and atrophy of the RPE, followed by progressive degeneration of photoreceptors [9]. These results support the hypothesis that oxidative damage of the RPE contributes to photoreceptor loss in AMD.

Endoplasmic reticulum (ER) is the primary intracellular organelle responsible for protein folding and maturation. Recent evidence suggests that disturbed homeostasis of the ER, or ER stress may also contribute to RPE damage and photoreceptor degeneration in AMD. Increased ER stress has been observed in several animal models of retinal degeneration such as P23H rhodopsin transgenic rats [10], RD1 mice [11], and light-induced retinal degeneration (LIRD) rats [12], accompanied by increased oxidative stress and apoptosis of photoreceptors [12]. This indicates a potential interaction between ER stress and oxidative stress in the process of retinal cell death, and this crosstalk is currently poorly understood. X-box-binding protein 1 (XBP1) is originally identified as a protein binding to the *cis*-acting X box region in the promoter of human major histocompatibility complex class II genes [13]. Recent studies have revealed that XBP1 is a master coordinator of the unfolded protein response (UPR) [14] and is involved in phospholipid biosynthesis, ER biogenesis, hepatogenesis, and cardiac myogenesis [15], [16], [17], [18]. During ER stress, XBP1 is activated by inositol-requiring enzyme 1 (IRE1), an endoribonuclease, through an unconventional splicing of XBP1 mRNA. The spliced mRNA is then translated into a new transcription factor (spliced XBP1, XBP1S), which binds to the ER stress-response element (ERSE) and upregulates its target genes such as ER chaperones and genes responsible for ER-associated degradation (ERAD) [14]. A recent study from Ryoo and colleagues demonstrates that depletion of XBP1 in Drosophila augments photoreceptor degeneration in ninaE (G69D)−/+, a Drosophila model for autosomal dominant retinitis pigmentosa (ADRP) [19], suggesting that XBP1 is essential for photoreceptor survival during ER stress. However, the role of XBP1 in the RPE has not been studied.

In the present study, we investigated the role of XBP1 in regulation of oxidative stress and apoptosis in RPE cells and its potential implication in AMD. Our results demonstrate that XBP1 activation in the RPE was suppressed by light stress, in parallel with decreased expression of ER chaperone and anti-oxidant genes, in a rodent model of retinal degeneration. Knockdown of XBP1 in RPE cells resulted in reduced expression of anti-oxidant genes accompanied by increased oxidative stress, decreased cell viability, and apoptosis. Consistently, conditional knockout of XBP1 in the RPE in mice led to exacerbated oxidative stress and ER stress in RPE cells, accompanied by RPE apoptosis and photoreceptor degeneration. These findings together provide strong evidence that XBP1 is a key player in coordinating the anti-oxidant system and ER stress response to protect the RPE against oxidative injury.

## Results

### Decreased XBP1 activation in the RPE in a light-induced retinal degeneration (LIRD) model

**Figure 1:**
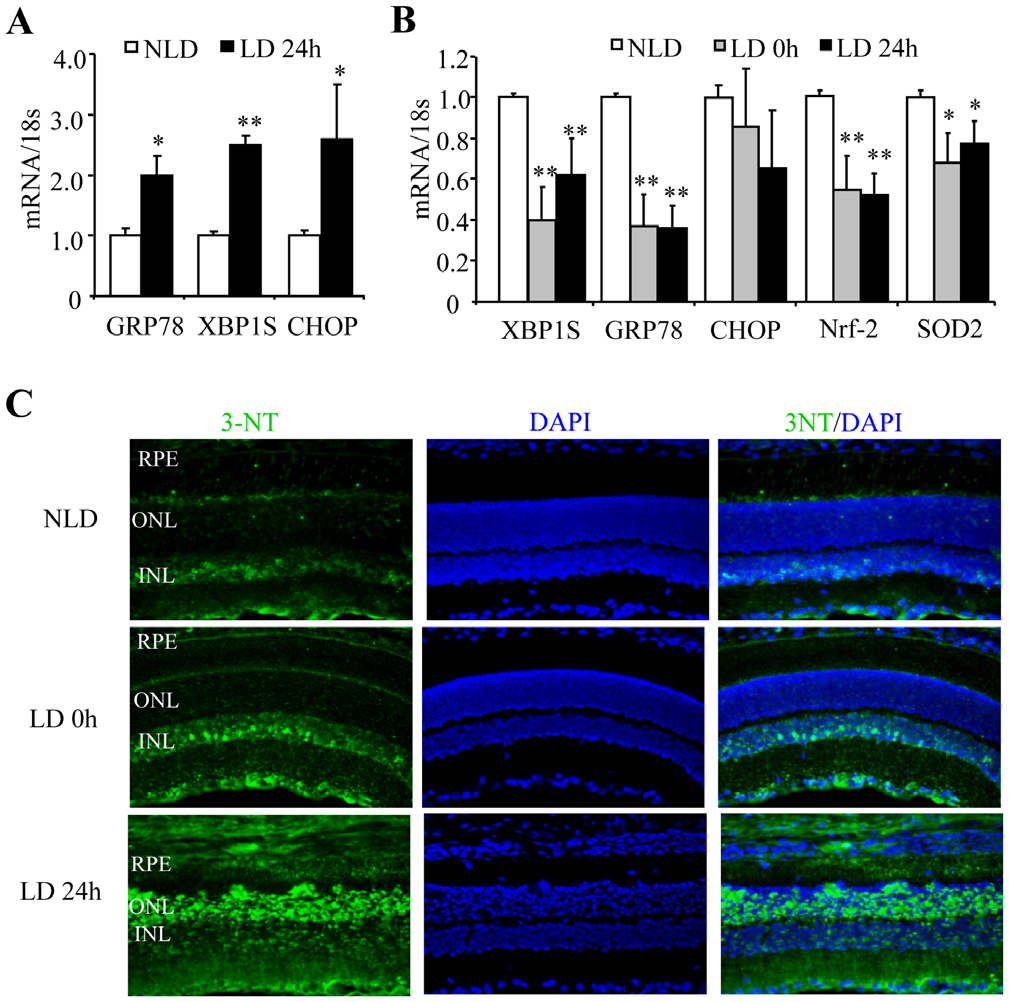
Reduced XBP1 activation correlates with decreased anti-oxidant genes in rat eyecups in light-induced retinal degeneration. Adult Sprague Dawley rats were exposed to 2700 lux light for 6 h. Retinas and eyecups were harvested immediately or 24 h after light stress. **A–B**). mRNA expression of ER stress response genes and anti-oxidant genes in the retina (**A**) and in the eyecup (**B**) was determined by real-time RT-PCR (mean ± SD, n = 3). *P<0.05, **P<0.01 vs. control. **C**). Cryosections of retina were immunostained with anti-3-nitrotyrosine (3-NT) antibody (green). DAPI was used for staining of cell nuclei (blue). NLD: non-light damage; LD: light damage. RPE, retinal pigment epithelium; ONL, outer nuclear layer; INL, inner nuclear layer. Representative images from 3 animals in each group.

Light-induced retinal damage in albino rodents is a commonly used model for studying retinal degenerative diseases [20]. Exposure to intensive bright light increases ROS generation, triggering photoreceptor cell death and RPE cell damage [21], [22]. Intriguingly, a recent study showed that several ER stress markers such as glucose-regulated protein-78 (GRP78), phosphorylated PKR-like endoplasmic reticulum kinase (PERK), and phosphorylated eukaryotic initiation factor 2α (eIF2α) were also elevated in the rat retina after light damage, indicating an induction of ER stress [23]. As XBP1 is a major regulator of ER stress response, we determined whether XBP1 is activated in LIRD. We found that the level of spliced (active) XBP1 in the retina was significantly increased after light stress, coincident with enhanced expression of retinal GRP78 and C/EBP homologous protein (CHOP), an ER stress-associated pro-apoptotic gene (Fig. 1A). In contrast, XBP1 activation and GRP78, but not CHOP, expression in the eyecup (i.e. a complex of RPE-choroid-sclera) were remarkably suppressed after intensive light stress (Fig. 1B). In parallel, expression of anti-oxidant gene Nrf2 and SOD2 in the eyecup were also significantly decreased (Fig. 1B). Similar changes were observed at 24 h after light stress. Moreover, the level of nitrotyrosine (3-NT), a commonly used marker for protein oxidation and oxidative stress, was increased in the RPE immediately after light stress (Fig. 1C), while intensive staining of 3-NT was observed in the outer nuclear layer (ONL) 24 h after light stress. These results suggest that disturbed XBP1 activation may correlate with decreased anti-oxidant gene expression and increased oxidative stress during RPE damage in LIRD.

### Knockdown of XBP1 results in decreased expression of anti-oxidant genes and sensitizes cells to oxidative damage in RPE cells

**Figure 2:**
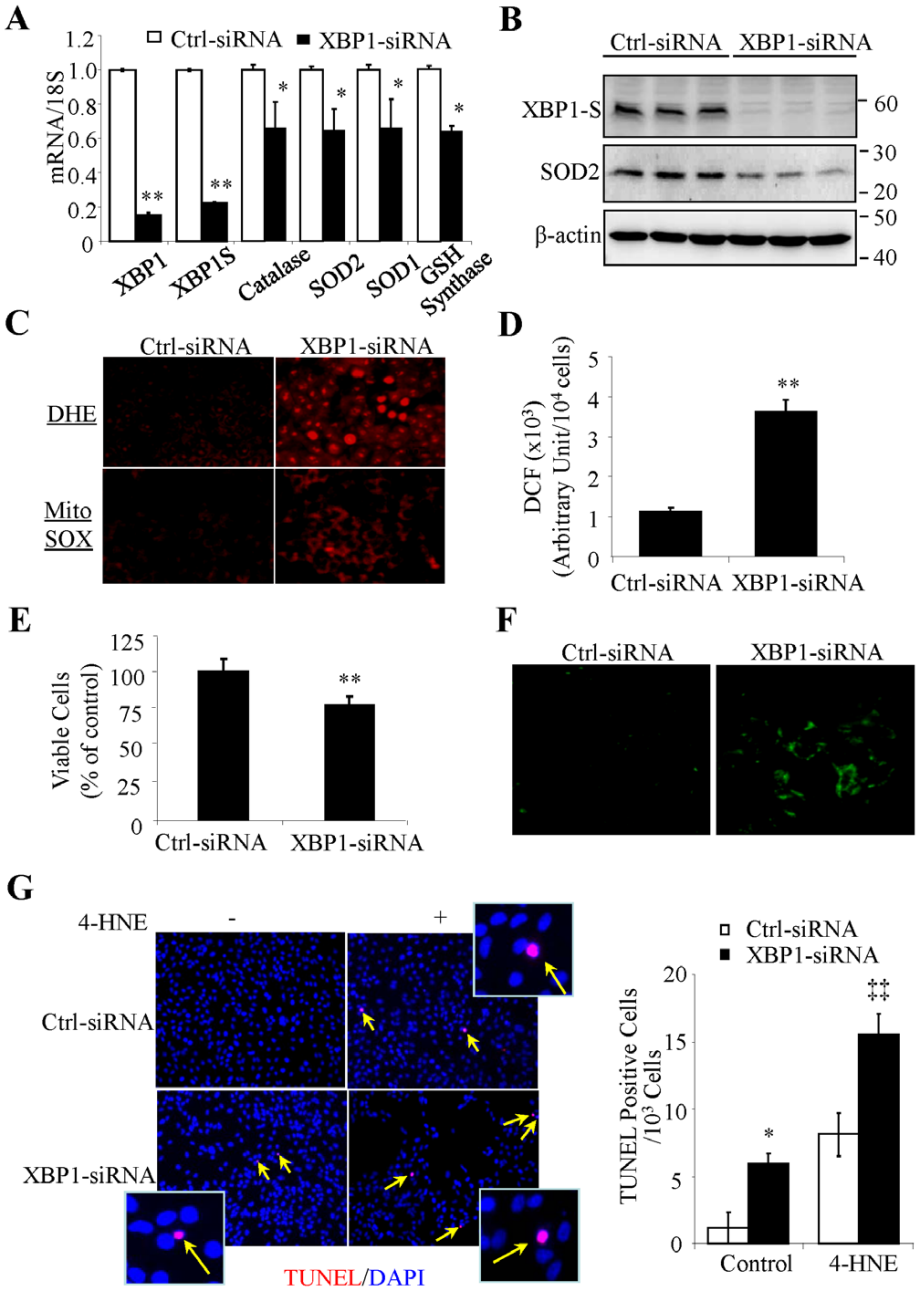
Knockdown of XBP1 down-regulates anti-oxidant genes in human RPE cells. Human RPE (ARPE-19) cells were transfected with XBP1 siRNA or control siRNA for 48 h. **A**). mRNA expression of antioxidant gene catalase, SOD2, SOD1, Nrf2, and GSH synthase was measured by real-time RT-PCR. Data were expressed as mean ± SD (n = 3 independent experiments). **B**). Protein levels of spliced XBP1and SOD2 were determined by Western blot analysis and semi-quantified by densitometry. **C**). Intracellular superoxide production was detected by Dihydroethidium (DHE) (upper panel). Mitochondrial superoxide level was analyzed by MitoSOX™ Red assay (lower panel). Representative images from 3 independent experiments are shown. **D**). Intracellular ROS generation was determined by DCF. The fluorescence density was quantified by using a fluorescence plate reader with wavelength of 485/535 nm (mean ± SD, n = 3). **E**). Cell viability was determined by MTT assay. The numbers of viable cells are expressed as % of control, averaged from 3 independent experiments (mean ± SD). **F**). Apoptosis was detected by Annexin V staining in ARPE-19 cells transfected with XBP1 siRNA or control siRNA. * *P*<0.05, ** *P*<0.01 vs. control siRNA. **G**). Transfected cells were exposed to 4-HNE (50 µM) for 24 h, apoptosis was detected by TUNEL assay. Left panels show representative images of TUNEL staining (red). Nuclei were stained with DAPI (blue). Right panel shows the quantitative results of apoptotic cells. * *P*<0.05 vs. control siRNA; ‡ *P*<0.01 vs. control siRNA+4-HNE.

We determined whether loss of XBP1 influences expression of anti-oxidant genes and oxidative stress in RPE cells. Down-regulation of XBP1 was successfully achieved by siRNA against human XBP1 in ARPE-19 cells, a human RPE cell line (Fig. 2A, 2B). Knockdown of XBP1 significantly decreased mRNA levels of SOD1, SOD2, catalase, and GSH synthase (Fig. 2A), and reduced the protein levels of SOD2 (Fig. 2B). Simultaneously, levels of intracellular superoxide (O_2_^−^) and ROS were significantly increased in XBP1-deficient cells (Fig. 2C, 2D). In addition, mitochondrial O_2_^−^ level, measured by mitochondria-targeted hydroethidine (MitoSOX red), was elevated in cells containing reduced XBP1 (Fig. 2C). This change correlated with the reduced level of SOD2 (Fig. 2B), a major mitochondria-located enzyme that catalyzes the dismutation of O_2_^−^ into H_2_O_2_. As excessive ROS can cause RPE cell death, we asked if depletion of XBP1 affects cell survival. We found that cell viability was decreased after XBP1-siRNA treatment (Fig. 2E), while apoptotic cells were increased as indicated by positive Annexin IV (Fig. 2F) and TUNEL staining (Fig. 2G). Furthermore, XBP1-deficient cells were more sensitive to oxidative stress. As shown in Fig. 2G, more apoptotic cells were observed in XBP1-siRNA-treated cells than in control cells when exposed to 4-HNE. These results suggest that XBP1 may be an essential survival factor that regulates the anti-oxidant defense in RPE cells, and that loss of XBP1 is sufficient to induce oxidative stress and RPE cell death.

### Generation of RPE-specific conditional XBP1 knockout mice

**Figure 3:**
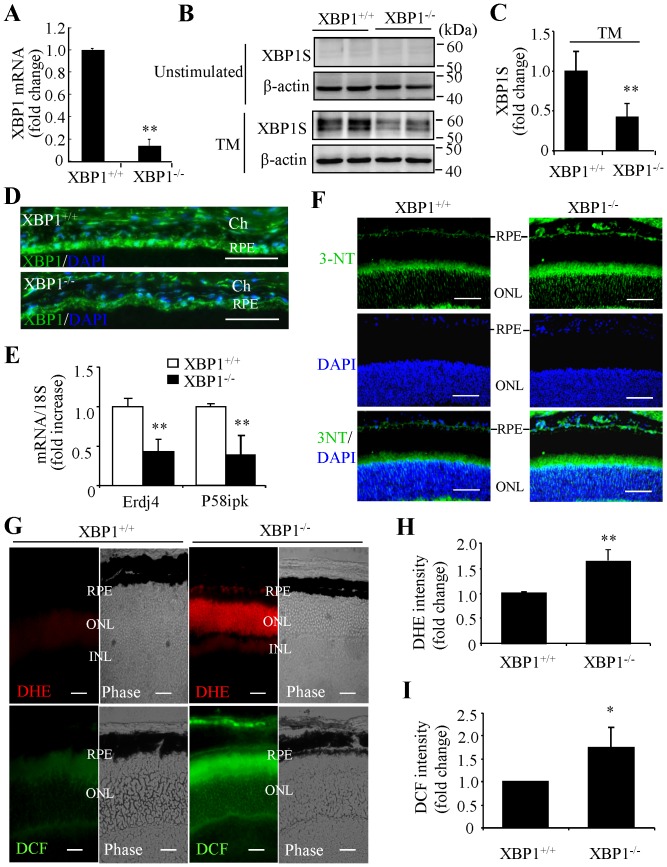
Increased oxidative stress in the RPE and photoreceptors in RPE-specific XBP1 KO mice. **A**). XBP1 expression in the RPE in XBP1 KO and littermate WT mice. The single layer of RPE cells was isolated as a sheet from 2-month-old XBP1 KO and littermate WT mice using dispase as described in the Methods section. Two RPE sheets from the same mouse were pooled and used for RNA isolation. XBP1 expression was measured by real-time RT-PCR. Results are expressed as mean ± SD (n = 6). **B–C**). Activation of XBP1 induced by ER stress in XBP1 KO and WT mice. Eyecups containing RPE, choroid, and sclera were incubated with 10 µg/ml tunicamycin for 6 h. Proteins were extract from the RPE by incubation of lysis buffer with the inner surface of the eyecups and subjected to Western blot analysis. Results show that spliced XBP1 (XBP1S) expression was undetectable in unstimulated eyecups (**B**, upper panel), but was markedly increased in WT mice compared to XBP1 KO mice (**B**, lower panel). XBP1S expression was quantified by densitometry (**C**) (mean ± SD, n = 6, **P<0.01). **D**). Immunostaining of XBP1 (green) in retinal cryosections from 2-month-old XBP1 KO and WT mice. Blue: nuclear staining with DAPI. **E**). mRNA expression of ERdj4 and P58IPK in the RPE was measured by real-time RT-PCR (mean ± SD, n = 6). **F**). Immunostaining of 3-NT (green) in retinal cryosections from XBP1 KO and WT mice. Blue: nuclear staining with DAPI. **G–J**). *In situ* dihydroethidium (DHE) and 2,7-CM-H_2_DCFDA (DCF) staining of fresh retinal cryosections from XBP1 KO and WT mice. Representative images from 4 animals in each group are shown in **G**. Note intensive staining of DHE (indicative of O_2_^−^) and DCF (indicative of ROS) in RPE and photoreceptor cells in XBP1 KO mice. **H–I**). Quantification of fluorescence intensity in the RPE layer shows a significant increase in O_2_^−^ and ROS levels in XBP1 KO mice (mean ± SD, n = 4). * *P*<0.05, ** *P*<0.01. Scale bar: 50 µm in D and F; 20 µm in G. RPE, retinal pigment epithelium; ONL, outer nuclear layer; INL, inner nuclear layer; Ch, choroid.

To further validate the role of endogenous XBP1 in RPE survival and function, we generated RPE-specific XBP1 knockout mouse – by breeding XBP1^flox^ mice [24] with inducible RPE-specific *cre* mice that specifically express Cre recombinase in the RPE [25]. The mice were backcrossed to C57/BL6 background for 5 generations. Cre expression was enhanced by feeding the nursing mother with doxycycline diet from P1-P21. To assess the knockout efficiency, RPE cells were isolated from XBP1 KO and control mice, and mRNA expression of XBP1was determined by real-time RT-PCR. As shown in Fig. 3A, XBP1 expression in RPE cells was significantly reduced to 11% in KO mice compared to littermate WT (XBP1^flox^) controls. We also examined the protein level of spliced XBP1 during ER stress. Considering the limited protein content in the single layer of RPE cells, we used eyecups, which contain the RPE-choroid-sclera complex. Eyecups from XBP1 KO or WT mice were incubated for 6 h with tunicamycin, a potent ER stress stimulator. Proteins were extracted and subjected to Western blot analysis. As expected, tunicamycin induced a significant greater increase in spliced XBP1 in the eyecups from WT mice compared to XBP1 KO mice (Fig. 3B, 3C). In addition, immunostaining revealed XBP1 expression in the nuclei in RPE cells in WT mice, which was significantly reduced in XBP1 KO mice (Fig. 3D). Furthermore, expression of ERdj4 and P58IPK, two major target genes of XBP1, was markedly reduced in XBP1 KO mice (Fig. 3E). These results suggest a successful down-regulation of XBP1 gene in the RPE of XBP1 KO mice. To determine whether loss of XBP1 in the RPE affects postnatal retinal development, we examined retinal morphology and function in XBP1 KO mice of age 1–2 months. We found that there were no detectable morphological and functional abnormalities in XBP1 KO mice compared to their littermate WT controls, suggesting that loss of XBP1 in the RPE had no impact on retinal development.

### Loss of XBP1 resulted in increased oxidative stress associated with decreased antioxidant gene expression in the RPE

**Figure 4:**
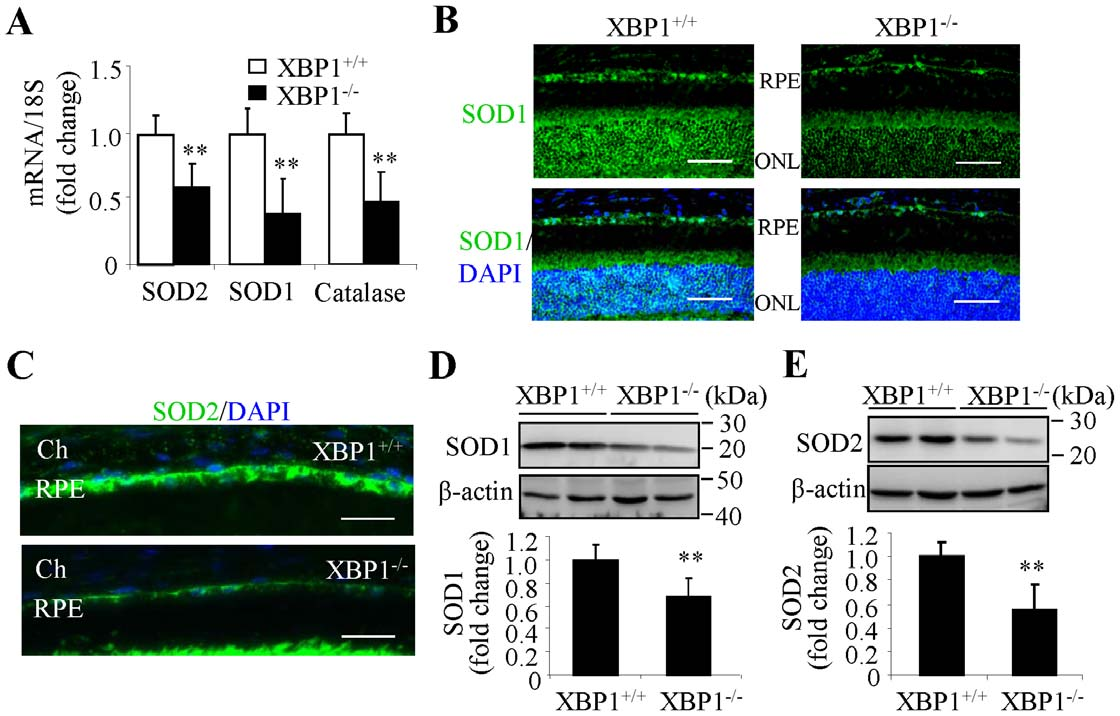
Decreased expression of antioxidant genes in the RPE in XBP1 KO mice. **A**). mRNA level of SOD2, SOD1, and catalase in the RPE was determined by real time RT-PCR (mean ± SD, n = 6, ** *P*<0.01). **B**). Retinal sections were stained with an anti-SOD1 antibody (green) and nuclei were stained with DAPI (blue). **C**). Immunostaining of SOD2 in the RPE in XBP1 KO and WT mice. Images represent 3 animals in each group. **D–E**). Western blot analysis revealed significant down-regulated expression of SOD1 and SOD2 in the eyecups of XBP1 KO mice. The protein levels of SOD1 and SOD2 were quantified using densitometry (mean ± SD, n = 6, **P<0.01). [scale bar, 50 µm (B); scale bar, 20 µm (C)]. RPE, retinal pigment epithelium; ONL, outer nuclear layer; Ch, choroid.

We next examined whether loss of XBP1 alters the status of oxidative stress in the RPE and retina, as determined by levels of 3-NT, O_2_^−^ and ROS in 2-month-old XBP1 KO and littermate WT mice. Immunohistochemistry showed that 3-NT was markedly increased in the RPE and the inner segment of photoreceptors in XBP1 KO mice compared to WT controls (Fig. 3F). In addition, O_2_^−^ and ROS levels in the RPE were elevated markedly in XBP1 KO mice (Fig. 3G, 3H, 3I). Strong signals of O_2_^−^ and ROS were also observed in the outer nuclear layer, which comprises the nuclei of photoreceptor cells. These results indicate that deficiency of XBP1 increases oxidative stress in the RPE and photoreceptors. To address whether this increased oxidative stress is associated with changes in anti-oxidant genes, we examined the expression of major O_2_^−^ and ROS scavenging enzymes (SOD1, SOD2 and catalase) in the RPE. Our results demonstrate that mRNA expression of SOD1, SOD2 and catalase was significantly decreased by 50% to 60% in XBP1 KO mice (Fig. 4A). Moreover, immunostaining revealed that SOD1 and SOD2 proteins were markedly reduced in the RPE of XBP1 KO mice (Fig. 4B, 4C). This reduction was further confirmed by Western blot analysis (Fig. 4D, 4E). These results corroborate the findings from our cell culture study that indicate that XBP1 is essential for expression of anti-oxidant genes in the RPE.

### Increased ER stress and apoptosis in the RPE in XBP1 KO mice

**Figure 5:**
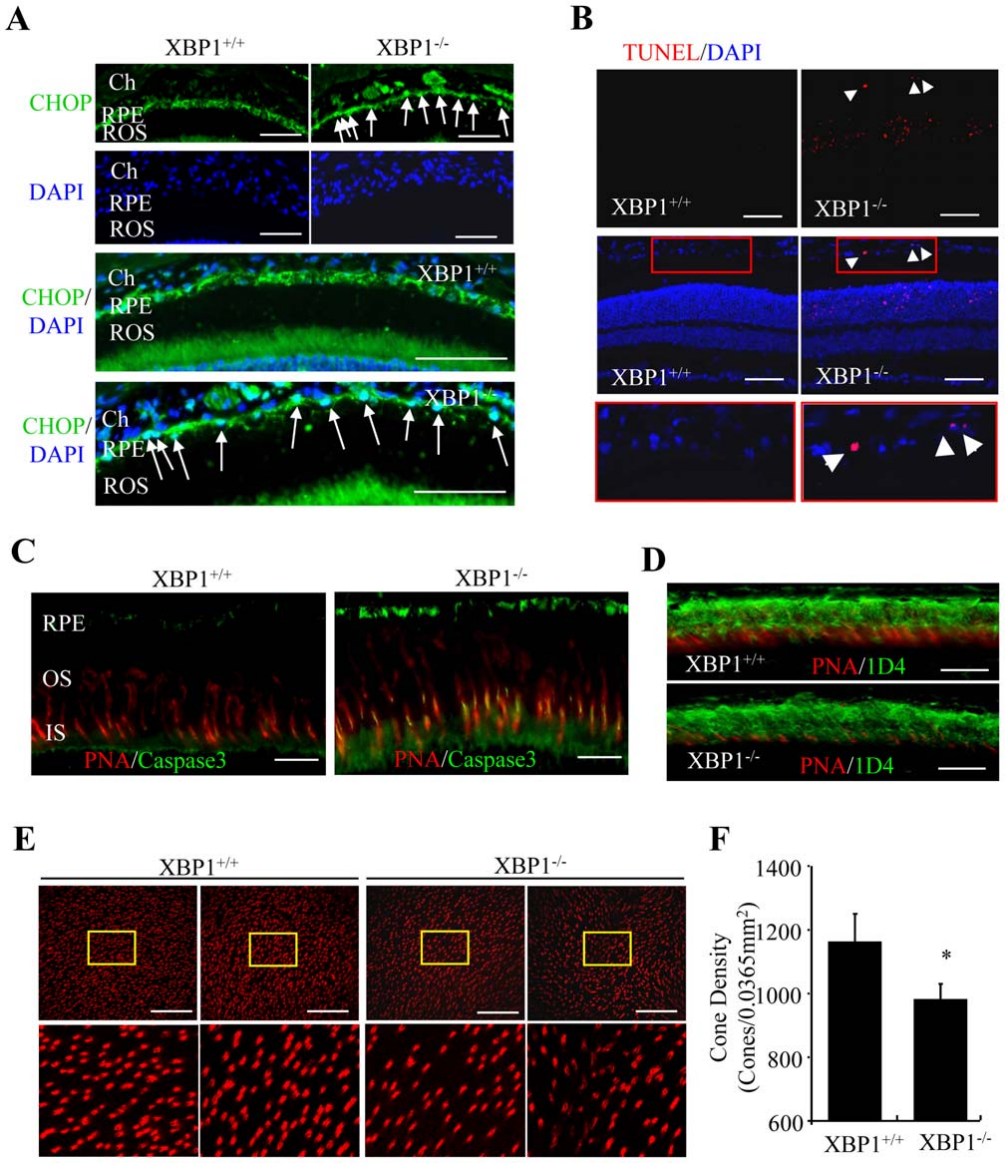
Increased RPE and photoreceptor apoptosis in XBP1 KO mice. **A**). Retina sections were stained with an anti-CHOP antibody and a secondary FITC-labeled antibody. Results show a markedly increased expression of CHOP (green) in RPE nuclei (white arrow) in XBP1 KO mice. DAPI was stained to locate cellular nuclei. **B**). TUNEL staining showed an increased apoptosis (red) in the RPE (white arrowhead) and ONL of XBP1 KO mice. **C**). Immunohistochemistry of caspase 3 (green) and peanut agglutinin (PNA, red) in the retina of 3-month-old WT and XBP1 KO mice. Note markedly increased staining of caspase 3 in cones and RPE cells in XBP1 KO mice. **D**). Retinal sections of 4-month-old mice were stained with anti-rhodopsin (1D4, green) antibody and PNA (red). **E**). Representative confocal images of retinal flat mounts in 4-month-old mice stained with PNA show reduced cone photoreceptors in XBP1 KO mice. **F**). Quantification of cone density in the retina. Cone numbers were counted and averaged from four 0.0365 mm^2^ fields in each retina. Results were obtained from 3 mice (mean ± SD). [scale bar: 50 µm (A, B, E), 20 µm (C, D)] ROS, retinal outer segment; RPE, retinal pigment epithelium; OS, outer segment; IS, inner segment; ONL, outer nuclear layer; INL, inner nuclear layer; GCL, ganglion cell layer.

**Figure 6:**
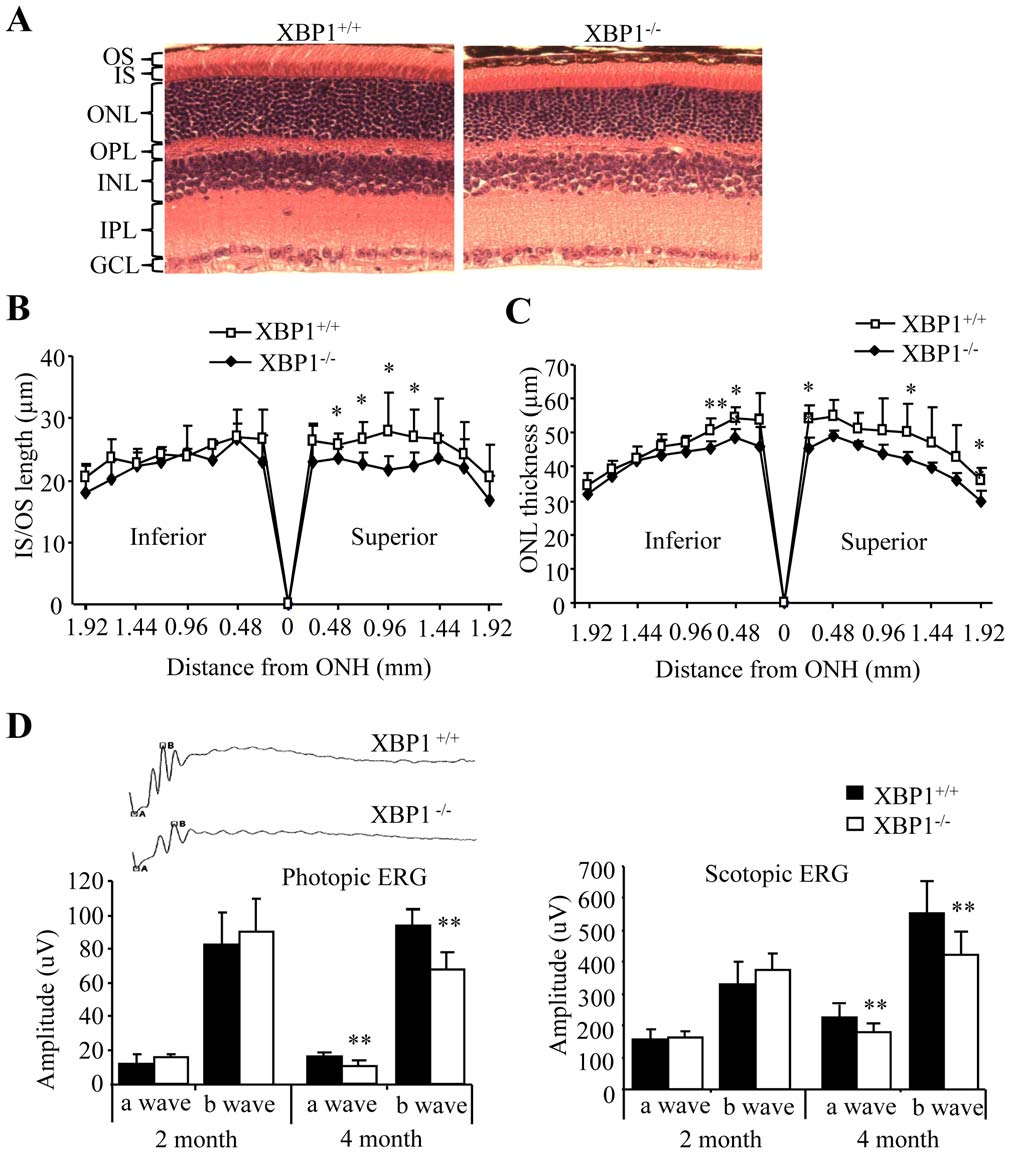
Altered retinal morphology and visual function in XBP1 KO mice. Histology and retinal function recorded by ERG in 4-month-old XBP1 KO and WT mice. **A**). Representative retinal sections. **B**). Mean length of photoreceptor IS/OS (mean ± SD, n = 5). **C**). Quantification of ONL thickness (mean ± SD, n = 7). **D**). ERG recordings show significantly decreased amplitude of a and b waves in both photopic and scotopic ERG in 4-month-old, but not in 2-month-old XBP1 KO mice.

To address whether XBP1 deletion in the RPE elicits ER stress, we examined the expression of CHOP, an ER stress-inducible pro-apoptotic gene, by immunofluorescence microscopy. In WT mice, CHOP was expressed at low level in cytoplasm of RPE cells (Fig. 5A). Depletion of XBP resulted in increased expression and translocation of CHOP from cytoplasm to the nucleus (Fig. 5A). As CHOP is a major mediator of ER stress-induced apoptosis, we further examined apoptosis markers using TUNEL assay. We found TUNEL-positive apoptotic cells in the RPE and outer nuclear layer in 3-month-old XBP1 KO mice, but not in their littermate WT controls (Fig. 5B). Interestingly, caspase-3 activation, indicated by expression of cleaved caspase-3, was observed in the RPE as well as in cone photoreceptors (co-localized with PNA staining) (Fig. 5C). In addition, the number of cone photoreceptors was decreased in 4-month-old XBP1 KO mice compared with littermate WT controls (Fig. 5D, E, F). In contrast, the changes in rod photoreceptors were relatively mild, which include the slightly shorter outer segments and thinner outer nuclear layer, in the KO mice (Figs. 6A, 6B, 6C). ERG analysis showed a modest but significant reduction in both a- and b-wave amplitudes in rod and cone ERGs in 4-month-old XBP1 KO mice, but not in younger animals (Fig. 6D). These results indicate that loss of XBP1 resulted in increased ER stress and apoptosis in the RPE, accompanied by mild photoreceptor degeneration and disturbed retinal function.

### Ultrastructural changes of RPE and photoreceptors in XBP1 KO mice

**Figure 7:**
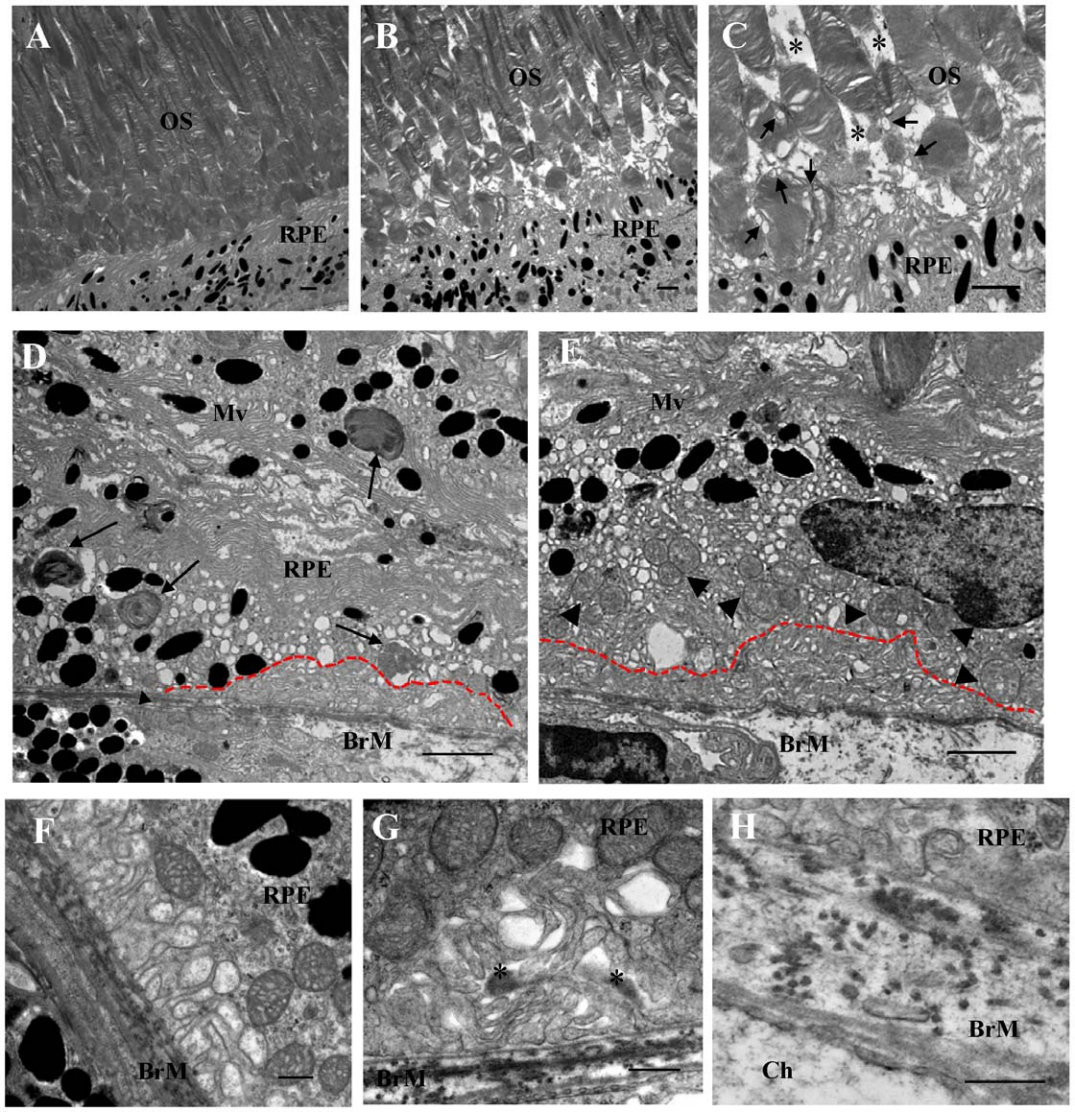
Electron microscopy of RPE and outer retina in old XBP1 KO mice. **A**). Electron micrograph of a 6-month-old WT mouse retina showing normal RPE and adjacent OS disk membranes. **B–C**). Highly disorganized OS and disrupted microvilla in a 6-month-old XBP1 KO mouse. Arrows indicate vesiculated OS disc membrane. Asterisks indicate atrophic area of OS. **D–E**). XBP1 KO mouse eyes display marked vacuolization in RPE and irregular basal infoldings outlined by a red dotted line. Arrows indicate autophagosomes containing undigested OS disc membranes. Arrowheads indicate swollen mitochondria in RPE. Note that the BrM is highly irregularly thickened in XBP1 KO mice. **F**). Ultrastructure of basal infoldings in a WT mouse. **G**). Disorganized basal infoldings in a XBP1 KO mouse. Asterisks indicate the accumulation of material between the RPE basal infoldings. **H**). Discontinuous collagen and elastin layers in the BrM in a XBP1 KO mouse. [Scale bar: 2 µm (A–D, H); scale bar: 500 nm (E–G)]. RPE, retinal pigment epithelium; OS, outer segment; Mv, microvilla; BrM, Bruch's membrane.

Finally, we determined how deletion of XBP1 affects the structure of the RPE and photoreceptors by examining the RPE and retinal ultrastructure in 6–10-month-old KO mice by transmission electron microscopy (TEM). The apical regions of the RPE in WT mice exhibited normal ultrastructure, with melanosomes and microvilli surrounding highly organized photoreceptor outer segments (Fig. 7A). In contrast, the RPE of XBP1 KO mice of same age displayed irregular, short, and fewer microvilla. Some photoreceptor outer segments were absent in the areas adjacent to the RPE (Fig. 7B, 7C). The remaining photoreceptors were disorientated with swollen, vacuolized, and disorganized disc membranes (Fig. 7B, 7C). In contrast, photoreceptors in the areas distal to the RPE appear normal, suggesting that the disruption of photoreceptor structure is associated with RPE damage.

Consistent with the immunostaining results showing apoptosis of the RPE in XBP1 KO mice (Fig. 6), TEM showed abnormal cellular ultrastructure compared to the WT mice. The KO mice exhibited loose cytoplasm with increased number of vacuoles, swollen mitochondria, and abnormal phagosomes that contain melanin and undigested outer segments (Fig. 7D, 7E). In addition, Bruch's membrane of KO mice was irregularly thickened with discontinuous collagen and elastin layers (Fig. 7D, 7E, 7F). In parallel, the tightly packed and highly organized structure of basal membrane infolding in normal RPE (Fig. 7G) was disrupted in the KO mice (Fig. 7D, 7E, 7H). However, we did not detect obvious drusen formation in XBP1 KO mice, examined by fundus examination, light microscopy, or TEM, although numerous lipofusin-like particles were observed in the RPE.

**Table 1:** Sequences of primers used in real-time RT-PCR.

Gene		forward	reverse	Suppl. Ref	
Human primers					
Catalase	ACTTTGAGGTCACACATGACATT		CTGAACCCGATTCTCCAGCA		PrimerBank ID 4557014a2
SOD2	AACCTCAGCCCTAACGGTG		AGCAGCAATTTGTAAGTGTCCC		Chou et al., 2008 [47]
SOD1	AGGGCATCATCAATTTCGAGC		GCCCACCGTGTTTTCTGGA		PrimerBank ID 4507149a1
GSS	CTCTACGGCTCACCCAATGC		TCGTCGGATCACATGGATGTT		PrimerBank ID 4504169a2
Murine primers					
XBP1s		GAGTCCGCAGCAGGTG	GTGTCAGAGTCCATGGGA		Gurzov et al.,2009 [48]
GRP78		TCATCGGACGCACTTGGAA	CAACCACCTTGAATGGCAAGA		Tirosh et al., 2006 [49]
CHOP		GTCCCTAGCTTGGCTGACAGA	TGGAGAGCGAGGGCTTTG		Tirosh et al., 2006 [49]
Erdj4		GCTGGCTGATCACATTCTGCT	GCCGTCCAACATGCCACTA		Tirosh et al., 2006 [49]
p58IPK		TCCTGGTGGACCTGCAGTACG	CTGCGAGTAATTTCTTCCCC		Tirosh et al., 2006 [49]
Nrf2		AGGACATGGAGCAAGTTTGG	TCCTCAAAACCATGAAGGAA		Mandal et al.,2009 [43]
SOD2		ATTAACGCGCAGATCATGCA	TGTCCCCCACCATTGAACTT		
SOD1		GACCTGGGCAATGTGACTGCTG	CACCAGTGTACGGCCAATGATG		
Catalase		GAACGAGGAGGAGAGGAAAC	TGAAATTCTTGACCGCTTTC		

PrimerBank: http://pga.mgh.harvard.edu/primerbank/.

## Discussion

XBP1 is a bZIP transcription factor that regulates multiple cellular processes, including ER biogenesis, protein folding, and lipogenesis [24]. Previous studies suggest that XBP1 is essential for cell survival in various cell types, e.g. plasma cells, hepatocytes [15], and intestinal epithelial Paneth cells [26], [27], but not in other cells [28]. For example, sustained expression of active XBP1 inhibits beta cell function and induces apoptosis in aortic endothelial cells [28]. The discrepancy among different cell types suggests that XBP1's effect on cell survival may be cell type-specific, possibly through regulation of distinct genes involved in cellular defense and apoptosis. In the present study, we report a novel function of XBP1 in regulation of oxidative stress and cell survival in the RPE. Deficiency of XBP1 in the RPE leads to apoptosis that correlates with decreased anti-oxidant gene expression and increased oxidative stress. Loss of XBP1 also induces ER stress, which may exaggerate RPE damage and apoptosis.

Oxidative stress has long been hypothesized to play a major role in the development of AMD [5], [6]. To cope with oxidative stress, the RPE hosts a potent endogenous defense system, which consists of enzymatic and non-enzymatic antioxidant genes including SODs, catalase, Nrf2, and many others. Several transgenic mice lines targeting the SOD oxidative stress-recovery pathways have been used to investigate the role of oxidative stress in AMD pathogenesis. Aged SOD1-deficient mice developed drusen, thickening of Bruch's membrane, marked RPE vacuolization, and destruction in IS/OS of retina [29], [30]. Lack of SOD2 gene in mouse germ line resulted in much greater and earlier retinal changes including photoreceptor degeneration compared to the SOD1 knockout mice [31]; however, these mice died before weaning due to dilated cardiomyopathy. To study the role of SOD2 in the RPE, Justilien and associates [9] injected an adeno-associated virus expressing ribozyme into the subretinal space of mice to knock down SOD2 in the RPE. They demonstrated that down-regulation of SOD2 resulted in increased oxidant damage of the RPE leading to progressive loss of photoreceptor cells and decreased retinal function. These findings argue that RPE injury may be the initial event in the pathogenesis of AMD. The retinal phenotype in our RPE-specific XBP1 KO mice further supports this notion. In our model, cone photoreceptors showed more remarkable changes than rod photoreceptors. The number of cones decreased by 33% in the KO mice, while the photoreceptor outer segments and the ONL thickness only showed modest decreases. This observation is consistent with the findings of a recent study by Wang and colleagues, which suggests that cones are more sensitive to RPE loss than rods [32]. In their study, systemic administration of reactive oxidant iodate via tail vein selectively damaged the RPE in the central retina of the mouse, followed by apoptosis of photoreceptors, preferentially cone photoreceptors. This phenomenon resembles the pathological changes of AMD in human, where the central loss of RPE is accompanied by cone photoreceptor degeneration in the macula.

Interestingly, in addition to the structural and functional changes in the photoreceptors, we observed disturbed b-wave in the ERG response from the RPE-XBP1 KO mice. Loss of ERG b-wave (41% decrease in b-wave vs. 33% decrease in a-wave) was also reported in mouse eyes lacking SOD2 only in the RPE [33]. ERG is a non-invasive method to record the activity of retinal neurons in animals as well as in human. Normal ERG response consists of two primary components: 1) a-wave that reflects the initiation of the visual signal in the photoreceptors; and 2) b-wave which is generated by depolarizing bipolar cells and, to a less extent, Müller cells and the third-order neurons such as amacrine and ganglion cells [34]. It remains unclear why oxidative damage of the RPE, which do not make physical contact with the second- or third-order retinal neurons, causes the dysfunction of these retinal cells. We speculate that disrupted barrier function of the RPE may alter the metabolic and immune homeostasis of the retina, resulting in reduced neuronal activity. In addition, RPE cells secret a variety of growth factors that support photoreceptor survival as well as other retinal neurons. Physically removal of RPE cells or in conditions of RPE dystrophy, Müller cells undergo degenerative changes, expressing high levels of GFAP and low levels of glutamine synthetase (GS) [35]. In contrast, in Royal College of Surgeons (RCS) rats with inherited retinal dystrophy, RPE transplantation significantly reduced GFAP expression in Müller cells and stabilized Müller cell activity [36]. These findings suggest that normal function of RPE cells is critical for maintaining photoreceptor survival and retinal neuronal activity.

Our data suggests that XBP1 plays an important role in regulation of anti-oxidant genes in the RPE. In our RPE-specific conditional XBP1 KO mice, both SOD1 and SOD2 expression in the RPE were significantly decreased by 30% and 45%, respectively. Catalase expression was also decreased in XBP1-deficient RPE cells. In contrast, O_2_^−^ and ROS levels were markedly increased in the RPE and photoreceptors, but not in the inner retina, accompanied by enhanced oxidative damage and apoptosis of RPE and photoreceptor cells. Recently, Liu and associates [37] reported that XBP1-deficient mouse embryonic fibroblasts exhibit decreased expression of anti-oxidant genes such as calalase, SOD1 and Trx1, and these cells more sensitive to oxidative damage induced by hydrogen peroxide. Interestingly, over-expression of unspliced XBP1, but not spliced XBP1, restores catalase expression in XBP1-deficient cells and alleviates ROS generation. In our study, both the *in vitro* and *in vivo* results indicate that XBP1 is essential for expression of antioxidant genes (SOD1, SOD2, and catalase). However, overexpression of spliced XBP1 failed to increase the expression of these anti-oxidant genes in RPE cells (Figure S1). These results coincide with the study by Liu and associates, suggesting that these genes may be indirectly regulated by XBP1. It is also possible that loss of XBP1 causes defects in other pathways important for redox gene transcription. This possibility is currently being investigated in our ongoing study. In addition, it is noteworthy that RPE cells are constantly exposed to high levels of free radicals generated by phototransduction. Complex mechanisms involving multiple transcription factors and signaling pathways may therefore be implicated in the regulation of anti-oxidant genes to ensure high capacity of the anti-oxidant defense in the RPE to accommodate excessive oxidant burden.

XBP1 is known as a key coordinator of the transcriptional program in response to ER stress. Activation of XBP1 upregulates ER chaperones and proteins involved in ERAD, including DnaJ/Hsp40-like genes, such as p58IPK, ERdj4, and HEDJ, as well as GRP78/BiP, EDEM, protein disulfide isomerase-P5, and ribosome-associated membrane protein 4 (RAMP4) [14]. These genes play pivotal roles in maintaining cellular viability during various stress conditions, such as nutrient deprivation, hypoxia, oxidative stress and inflammation [38]. Inactivation of XBP1 sensitizes cells to apoptosis after chronic or irreversible oxidative stress [37]. Similarly, knocking down XBP1 or IRE1α enhances cell death under conditions of chronic ER stress [39]. Intriguingly, a body of recent studies demonstrate that expression and activity of XBP1 and several ER chaperones, such as GRP78, PDI, ERp57 and p58IPK, are significantly decreased in tissues of old vs. young animals [40], [41]. In contrast, expression of the pro-apoptotic factor CHOP/GADD153 was induced and caspase-12 was activated in stressed aged rats but not in young animals [41]. These results suggest that declined levels of XBP1 and its downstream ER chaperones, and the resulting inability of the ER to cope with cellular stresses, may contribute to age-related cell death and dysfunction. In the present study, we found that exposure of rats to excessive light induced ER stress and subsequent XBP1 activation in the retina. However, light damage selectively inhibited XBP1 activation and reduced GRP78 expression in the RPE in the same animals. The mechanisms underlying these distinct responses to light stress in the RPE and in the retina are currently unknown. Interestingly, differential regulation of several transcripts in the RPE and in the retina was reported by Rattner and colleagues [42]. They demonstrated that a set of genes encoding proteins involved in the visual cycle such as Rpe65, Lrat, cellular retinaldehyde-binding protein (Cralbp), retinol-binding protein 1 (Rbp1), Rdh5 and Rdh10 were downregulated in the RPE in response to light damage. In contrast, Rdh10 and Rbp1 transcripts were increased in the inner nuclear layer of the retina. This observation indicates that the visual cycle is suppressed after light damage and this response may be photoprotective [42]. Whether XBP1 and ER chaperones are implicated in the regulation of ER-located visual cycle proteins remains to be investigated. Nevertheless, our study suggests that disrupted XBP1 activation may sensitize RPE cells to oxidative damage by down-regulating anti-oxidant genes, increasing oxidative stress and ER stress, and ultimately leading to cell death of the RPE and photoreceptors.

Taken together, our data demonstrate that loss of XBP1 or declined activation of XBP1 in the RPE leads to reduced anti-oxidant gene expression, increased oxidative stress and ER stress, resulting in RPE and photoreceptor apoptosis and cell death. These results strongly suggest an essential role of XBP1, which may act as a central coordinator of the cellular defense system, in maintaining the redox homeostasis and normal ER function in RPE cells. Future studies will be necessary to investigate whether overexpressing active XBP1 could prevent the decline of anti-oxidant capacity in aged RPE and to identify small molecules that can enhance XBP1 activity to protect RPE cells from oxidative damage during AMD.

## Materials and Methods

### Animals

All animal procedures were approved by the Institutional Animal Care and Use Committees at the University of Oklahoma Health Sciences Center and Dean A. McGee Eye Institute (protocols 08-086 and 10-038), and conformed to the guidelines of the ARVO statements for the “Use of Animals in Ophthalmic and Vision Research". XBP1^flox^ mice were generated by targeting *loxP* sites to introns flanking exon 2 of XBP1 gene [26]. To generate RPE-specific XBP1 knockout mice, XBP1^flox^ mice were bred with inducible RPE-specific *cre* transgenic mice, which express Cre recombinase in the RPE [25]. Mice were backcrossed to C57/BL6 background for 5 generations. Littermates were used as control in all experiments. Sprague Dawley (SD) rat breeders were purchased from Harlan and bred and maintained at dim (5 lux, 7:00 AM to 7:00 PM)/dark cyclic light.

### Bright-light induced retinal damage in rats

SD rats were used in light damage experiment as described previously [43]. Briefly, rats were placed individually in clear plastic cages with wire tops. The right eye of each rat was covered with a black-painted polypropylene eye cap attached to the facial skin (No. 454; Loctite Corp., Hartford, CT) to serve as a non-light-damaged control (covered eye, NLD). The left eye was uncovered and considered as the light-damaged eye (uncovered eye, LD). Rats were exposed to 2700 lux white cool light for 6 h, and euthanized immediately after light exposure or returned to a dim cyclic light environment for 24 h. Retinas and eyecups were harvested and processed for experiments.

### Electroretinography (ERG)

Retinal function was assessed by ERG in XBP1 KO and WT mice using an Espion E^2^ System (Diagnosys, Lowell, MA). Briefly, mice were overnight dark-adapted, anesthetized, and pupils dilated with 1% atropine and 1% cyclopentolate. A reference electrode was positioned in the cheek mucosa and another in the tail. ERG responses from both eyes were recorded simultaneously with gold electrodes placed on the cornea. Two flash intensities, 1000 and 2000 cd.s/m^2^, were used. The a-wave and b-wave amplitudes from each eye were determined and averaged for comparison of retinal function.

### Immunohistochemisty

Retinal frozen and paraffin sections were prepared as described previously [44]. Antigen retrieval was performed in paraffin sections by heating the sections in 10 mM sodium citrate buffer (pH 6.0) at a sub-boiling temperature for 30 min followed by cooling at 4°C. Retinal sections were immunostained using anti-XBP1 (1∶100), anti-Nrf2 (1∶100), anti-CHOP (1∶100) (Santa Cruz Biotechnology, CA), anti- SOD1 (1∶200; Abcam, Cambridge, MA), anti-SOD2 (1∶250; Assay Designs, MI), anti-cleaved-caspase-3 (1∶100; Cell Signalling Technology, Boston, MA), and anti-rhodopsin (1D4, 1∶200; Chemicon international, IL) antibodies. Antibodies were visualized using Cy3-conjugated secondary antibody (Jackson Immunoresearch Laboratories, West Grove, PA) or biotinylated matching secondary antibody and FITC avidin (Vector Laboratories, Burlingame, CA). For staining of cone photoreceptors, retinal sections or flatmounts were stained with rhodamine peanut agglutinin (Vector Laboratories, Burlingame, CA). The fluorescence was visualized under an Olympus AX70 microscope (Olympus, Japan).

### Human RPE cell culture and siRNA transfection

Human RPE (ARPE-19) cells were obtained from American Type Culture Collection (ATCC, Manassas, VA) and maintained in Dulbecco's Modification of Eagle's Medium/Ham's F12 50/50 Mix containing 10% fetal bovine serum and 1% antibiotic/antimycotic solution. ARPE-19 cells in 6-well plate with 40% to 50% confluence were transfected with XBP1 siRNA or control siRNA (Santa Cruz Biotechnology, Santa Cruz, CA) using Lipofectamine 2000 (Invitrogen, Carlsbad, CA) following manufacturer's instruction.

### Transduction of adenoviruses in ARPE-19 cells

ARPE-19 cells at 50%–60% confluence were transduced with adenoviruses expressing spliced XBP1 as described previously [45]. Adenovirus expressing LacZ was used as control. After 24 h transduction, cells were quiescent overnight with serum free DMEM/F12 medium. Cells were harvested and processed for Western blot analysis.

### MTT cell viability assay

After siRNA transfection for 48 h, cell viability of ARPE-19 cells was measured by 3-[4,5-yl]-2,5-diphenyltetrazolium bromide (MTT) assay kit (Trevigen, Gaithersburg, MD) following manufacturer's instructions. Briefly, 10 µl MTT solution (Cat# 4890-25-01) was added to the cells in 100 µl medium. After an incubation period of 4 h, 100 µl detergent reagent (Cat# 4890-25-02) was added and cells were incubated overnight at 37°C. Absorbance was measured at 570 nm in a microplate reader.

### Apoptosis assays

Annexin V-FITC and TUNEL apoptosis assays were used to detect apoptosis in cultured ARPE-19 cells or on cryosections of mouse eyes. Briefly, after transfection with siRNA for 48 h, ARPE-19 cells were labeled with Alexa fluor 488 annexin V (Invitrogen, Carlsbad, CA) following manufacturer's recommendations and examined by fluorescence microscopy (Olympus AX70). TUNEL assay was performed using the In Situ Cell Death Detection TMR red kit (Roche Diagnostic) in ARPE-19 cells or on eye sections. For staining of sections, samples were permeabilized for 2 min in cold PBS containing 0.1%Triton X-100, and then incubated at 37°C in TUNEL reaction mix containing nucleotides and terminal deoxynucleotidyl transferase (TdT). Incubation without TdT enzyme was conducted as a negative control.

### Detection of ROS generation in RPE cells

Intracellular and mitochondrial superoxide production was indicated with dihydroethidium (DHE) and MitoSOX™ Red (Invitrogen, Carlsbad, CA), respectively. Intracellular hydrogen peroxide generation was determined by 2,7-CM-H_2_DCFDA (DCF, Invitrogen, Carlsbad, CA) as described before [46]. Briefly, after transfection with XBP1 siRNA or control siRNA for 48 h, ARPE-19 cells were incubated with 5 µM DHE for 30 minutes or 10 µM MitoSOX™ for 10 min in phenol-red free DMEM/F12. For DCF staining, the ARPE-19 cells were incubated with 10 µM DHDCF for 45 min. Fluorescence intensity was quantified by microplate reader (Perkin Elmer, Waltham, MA) with wavelength of excitation at 485 nm and emission at 535 nm.

### *In situ* detection of ROS generation in the RPE and retina

*In situ* ROS and superoxide generation in the retina and the RPE was determined as described previously [46]. Briefly, freshly prepared unfixed retinal cryostat sections were incubated with 10 µM 2,7-CM-H_2_DCFDA for 60 minutes or 0.625 µM DHE for 20 minutes at 37°C. The fluorescence was visualized under an Olympus AX70 microscope. Fluorescence intensity was quantified using Adobe Photoshop CS (Adobe Systems, San Jose, CA).

### Measurements of cone photoreceptor density

Mouse eyeballs were fixed in 2% paraformaldehyde for 1 h. Retinas were carefully dissected, blocked with 10% normal horse serum in PBS with 0.5% Triton X-100, and incubated with rhodamine-conjugated peanut agglutinin (PNA, 1∶500) for 1 h. Retinal flatmount was prepared with photoreceptors facing upward and examined under a FluoView FV500 confocal laser scanning microscope (Olympus). The number of cones was determined in four randomly selected high magnification fields adjacent to the optic nerve head.

### Histology and morphometric analysis

Paraffin sections of eyeballs were cut along the vertical meridian through the optic nerve head. Sections were dewaxed, rehydrated, and stained with hematoxylin and eosin. The ONL thickness and IS/OS length were measured under light microscopy. Measurements were taken every 240 µm from the optic nerve head to the peripheral retina on both the superior and inferior portions of the retina as described previously [43].

### Real-time RT-PCR

For ARPE-19 cells, total RNA was extracted using an E.Z.N.A. total RNA kit I (Omega bio-tek, Georgia, GA) following the manufacturer's instructions, and 1 µg RNA was used to synthesize cDNA. For mice and rats, total RNA was isolated from eyecups using TRIzol reagent (Invitrogen). A Maxima First Strand cDNA synthesis kit containing oligo(dT) and random hexamer primers (Fermentas) was used for cDNA synthesis. Real-time RT-PCR was performed using SYBR® Green PCR Master Mix (Bio-Rad Laboratories, Hercules, CA). Primers used for analysis are listed in Table 1. The mRNA levels of target genes were normalized by 18 s ribosomal RNA.

### Western blot analysis

Cells or tissues were lysed in radioimmunoprecipitation assay lysis buffer. For RPE extracts, two RPE/choroid preparations from each mouse were pooled and incubated in the lysis buffer for 30 min and then centrifuged at 13,300 rpm for 10 min. Twenty-five micrograms of protein were separated on SDS-PAGE and electro-transferred to nitrocellular membranes. After blocking, membranes were blotted overnight at 4°C with following primary antibodies: anti-XBP1 (1∶500), anti-Nrf2 (1∶500) (Santa Cruz Biotechnology), anti-SOD1 (1∶1,000; Abcam), anti-SOD2 (1∶4,000; Assay Designs, MI), anti-catalase (1∶2000, Sigma-Aldrich, St. Louis, MO), and anti-β-actin (1∶5,000, Abcam). After incubation with HRP-conjugated secondary antibodies, membranes were developed with enhanced chemiluminescence substrate using Bio Imaging System (Syngene, Frederick, MD). The bands were semi-quantified using densitometry.

### Statistical Analysis

The quantitative data were expressed as mean ± SD. Statistical analyses were performed using unpaired Student's *t*-test when comparing two groups and one-way analysis of variance (ANOVA) with Bonferroni's multiple comparison test for three groups or more. Statistical differences were considered significant at a *P* value of less than 0.05.

## Supporting Information

Figure S1 **Expression of anti-oxidant genes in ARPE-19 cells overexpressing spliced XBP1.** Cells were transduced with adenoviruses expressing spliced XBP1 (Ad-XBP1) or control adenoviruses (Ad-LacZ) at MOI of 10 for 24 h. Protein levels of anti-oxidant genes were examined by Western blot analysis.
(TIFF)
