# Supplementary figures and images for: X-Box Binding Protein 1 Is Essential for the Anti-Oxidant Defense and Cell Survival in the Retinal Pigment Epithelium
Source: PLoS One. 2012 Jun 8;7(6):e38616. doi: 10.1371/journal.pone.0038616 (PMC3371004; doi:10.1371/journal.pone.0038616)

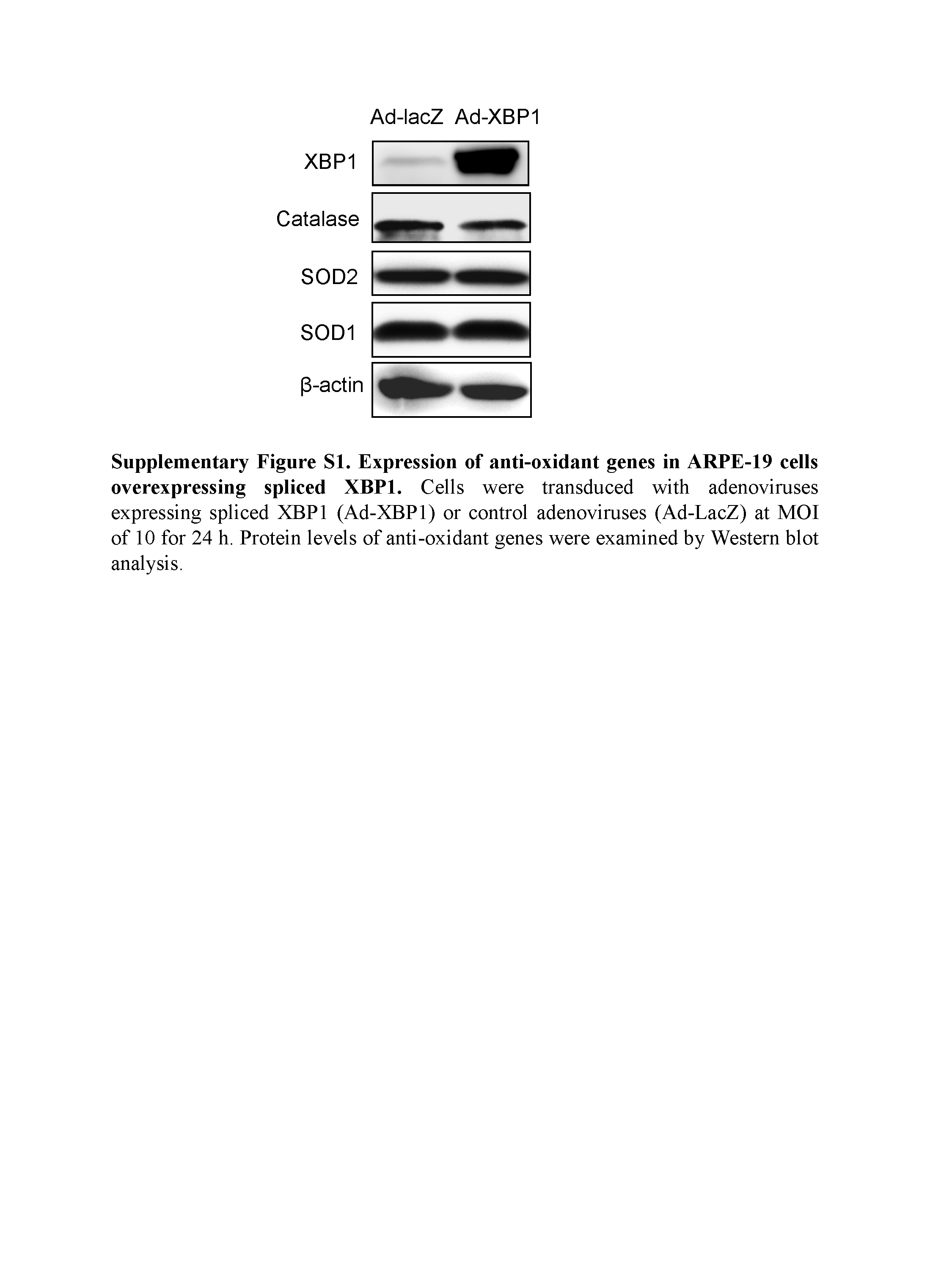

Supplement: Figure S1 — Expression of anti-oxidant genes in ARPE-19 cells overexpressing spliced XBP1. Cells were transduced with adenoviruses expressing spliced XBP1 (Ad-XBP1) or control adenoviruses (Ad-LacZ) at MOI of 10 for 24 h. Protein levels of anti-oxidant genes were examined by Western blot analysis. (TIFF) [file pone.0038616.s001.tiff]
